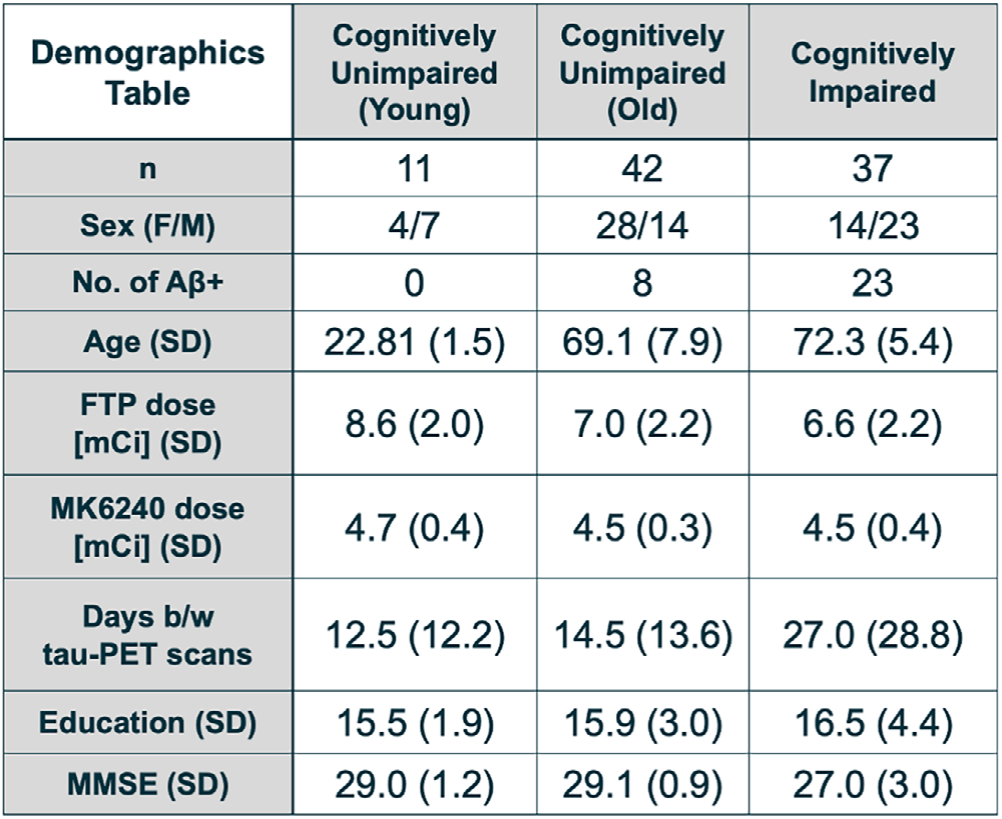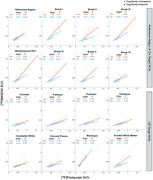# Comparison of [^18^F]MK6240 and [^18^F]Flortaucipir standardized uptake values (SUVs): the HEAD study

**DOI:** 10.1002/alz.093894

**Published:** 2025-01-09

**Authors:** Cécile Tissot, Hsin‐Yeh Tsai, Dana Tudorascu, Firoza Z Lussier, Nesrine Rahmouni, Stijn Servaes, Joseph Therriault, Jenna Stevenson, Brian A. Gordon, Belen Pascual, Val J. Lowe, David N. Soleimani‐Meigooni, Hwamee Oh, William E Klunk, Pedro Rosa‐Neto, William J. Jagust, Tharick Ali Pascoal, Suzanne L. Baker

**Affiliations:** ^1^ Lawrence Berkeley National Laboratory, Berkeley, CA USA; ^2^ Lawrence Berkeley National Lab, Berkeley, CA USA; ^3^ University of Pittsburgh, Pittsburgh, PA USA; ^4^ McGill University, Montreal, QC Canada; ^5^ Washington University in St. Louis School of Medicine, St. Louis, MO USA; ^6^ Houston Methodist Research Institute, Houston, TX USA; ^7^ Mayo Clinic, Rochester, MN USA; ^8^ Memory and Aging Center, Weill Institute for Neurosciences, University of California, San Francisco, San Francisco, CA USA; ^9^ Brown University, Providence, RI USA; ^10^ University of California, Berkeley, Berkeley, CA USA

## Abstract

**Background:**

Differences between on‐ and off‐target retention characteristics between [18F]MK6240 and [18F]Flortaucipir (FTP) complicate the harmonization across tracers. Our objective here was to separate the impact of the reference region by evaluating correlations between [18F]MK6240 (MK) and [18F]FTP standard uptake values (SUVs).

**Method:**

Participants (Figure 1, n=90) received an amyloid‐β (Aβ) PET scan ([11C]PIB or [18F]NAV4694) and two tau‐PET scans: [18F]MK (90‐110 minutes post‐injection) and [18F]FTP (80‐100 minutes post‐injection). For all PET scans, the individual frames were realigned, averaged, and co‐registered to a Freesurfer 7.1‐segmented T1‐MPRAGE. SUVs were calculated in the reference region (inferior cerebellar gray), on‐target regions (entorhinal cortex, hippocampus, Braak III, Braak IV, Braak V, Braak VI) and off‐target regions (caudate, pallidum, putamen, thalamus, cerebellar white matter, choroid plexus, meninges, eroded white matter). We examined correlations between [18F]MK and [18F]FTP SUV values as a function of Aβ status, as well as their associations with age.

**Result:**

All correlations (Figure 2) were significant at p<0.001. The interaction with Aβ status was significant for hippocampus, BraakIII‐V and the metatemporal region. In Aβ+ participants, [18F]FTP and [18F]MK6240 have similar SUVs in on‐target regions with a slope close to 1 except for in hippocampus and Braak VI. However, [18F]FTP SUVs, on average, were greater than [18F]MK SUVs in Aβ+ and Aβ‐ subjects in the reference region and all off‐target regions except for meninges, and in Aβ‐ subjects in the on‐target regions. Age correlated with [18F]FTP SUVs in Aβ‐ subjects in all regions (except the reference region, Braak VI and meninges) and [18F]MK SUVs in putamen and pallidum, but this correlation was only significant (p<0.01) when young and old subjects were included.

**Conclusion:**

Despite [18F]MK’s higher affinity for tau and SUVR dynamic range being twice that of [18F]FTP, the SUVs in on‐target regions are similar. The higher [18F]FTP signal in the reference region appears to be the contributing factor to the lower dynamic range. The higher [18F]FTP signal in on‐target regions for Aβ‐ subjects may reflect the presence of off‐target signals in these subjects. Taking off‐target signals into account should improve correlations between the two tracers, especially when measuring the earliest deposition of tau.